# Sherman S Coleman: Beloved Surgeon, Educator, and Friend

**DOI:** 10.7759/cureus.73547

**Published:** 2024-11-12

**Authors:** Ndidi C Njoku, Kristen L Carroll

**Affiliations:** 1 Orthopaedic Surgery, Howard University College of Medicine, Washington, DC, USA; 2 Orthopaedic Surgery, University of Utah, Salt Lake City, USA; 3 Orthopaedic Surgery, Shriners Children’s Salt Lake City, Salt Lake City, USA

**Keywords:** clinical mentor, historical vignette, orthopedic surgery, passionate teacher and mentor, professional mentor

## Abstract

Dr. Sherman S. Coleman was a superior surgeon, a pioneer of multiple orthopedic organizations, and a remarkable educator of surgical principles. He served as chair of orthopedic surgery at the University of Utah for 25 years, was an organizer and the president of the Pediatric Orthopedic Society of North America, and was a renowned physician-scientist and author of 88 journal articles, two books, and 16 book chapters. He was a beloved physician mentor and friend to scores of patients, trainees, and colleagues, with a long and fruitful career. This article reviews his path in orthopedics with the help of fellow physicians and most importantly his beautiful wife, Jane.

## Introduction and background

In June 2024, Dr. Kristen Carroll drove to the home of her mentor, Dr. Coleman, to meet with his widow, Jane. Dr. Coleman’s wife is 99, lives alone in their house, and is as gracious and lovely as ever. Long ago, Dr. Carroll remembered telling Dr. Coleman, “You are my professional mentor, but Jane is my personal one.” It’s still true: Jane Coleman defines what a lady should be. When Dr. Carroll arrived, she asked Jane, “What would Dr. Coleman want written about him?” She gazes at the garden and replies: “Well, he loved orthopedics, but even more, he loved teaching orthopedics. And his patients - he loved his patients. He also always wanted to get to the bottom of things, so research was a love, too.”

The purpose of this article is to celebrate the achievements of Dr. Sherman Coleman while providing a glimpse into his life and the lasting impact of his character. Dr. Coleman was a well-known orthopedic surgeon who dedicated much of his practice to the University of Utah (UU) specializing in pediatric tumors and skeletal deformities [1]. He was respected and beloved not only by his patients but also by his colleagues worldwide and by the many mentees he inspired throughout his career.

## Review

Sherman Smoot Coleman was born and raised in Utah as the great-grandson of a former Pioneer of the State, Abraham D. Smoot. Smoot served as the second mayor of Salt Lake City and the first mayor of Provo, the home of Brigham Young University (BYU), which he helped build [2].

**Figure 1:**
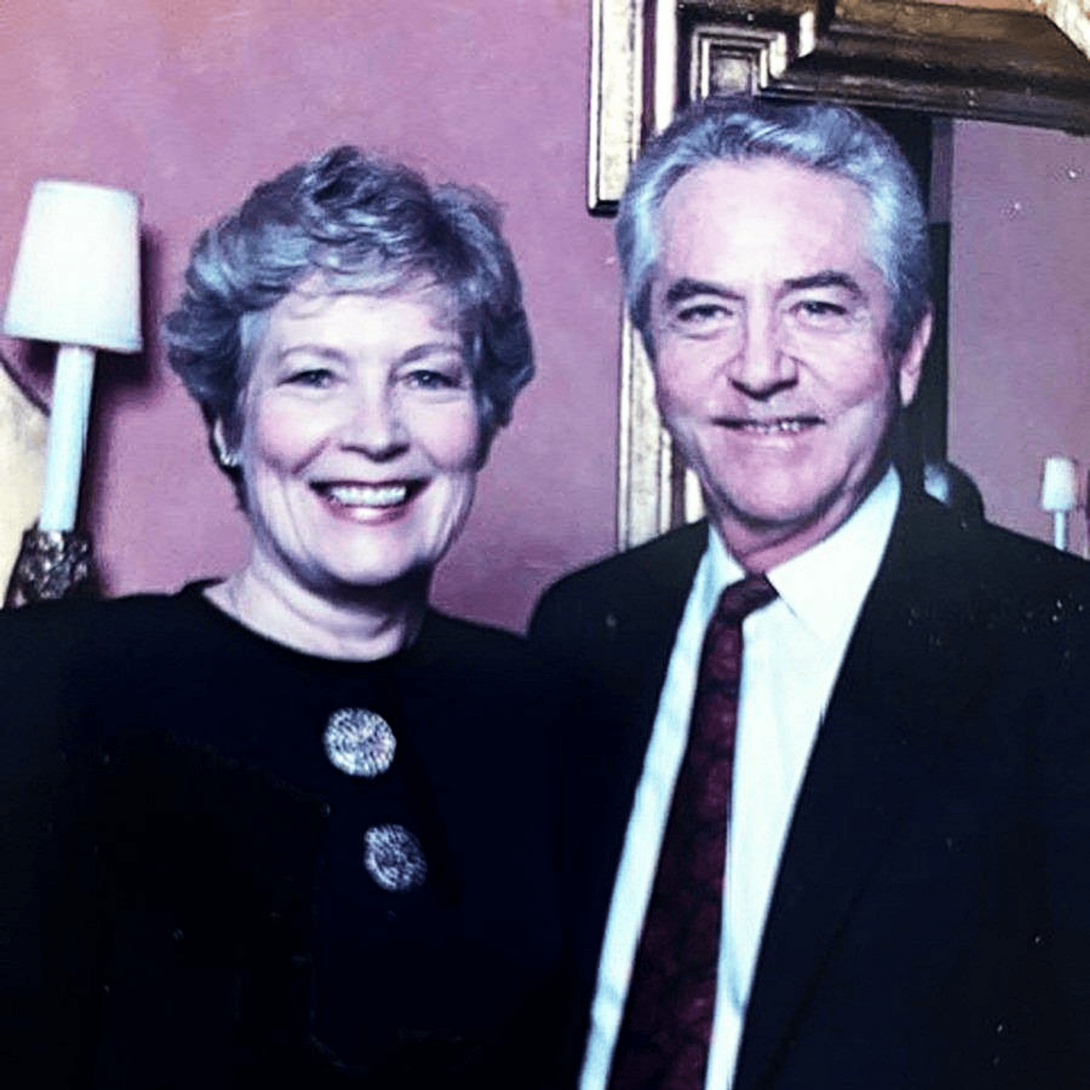
Dr. Sherman Coleman and his wife, Jane Coleman. Permission was obtained from the Coleman family.

Dr. Coleman was born in Provo, Utah, the son of a brilliant attorney, Jacob, and his wife, Alice. Even with four highly gifted siblings, Sherman quickly showed his acumen in learning, education, and athletics. His premedical undergraduate studies at BYU were interrupted by a two-year term in the Navy during World War II. After he came home, he completed his undergraduate degree in 1946 at the University of Chicago where he met and married Jane Dalenberg, a nursing student at the time (Figure 1). He obtained his medical degree at Northwestern University School of Medicine in 1948 [3,4].

Fascinated with education, Coleman stayed in Chicago to earn a master’s degree from Northwestern in surgical training. He then returned to active duty in the Korean War as a Naval officer lieutenant. After his Navy commitment, he completed his medical internship training at Los Angeles Hospital and then returned to Northwestern as a resident in orthopedics. In 1955, he completed his resident surgical training and joined the staff at Northwestern [2-4].

In 1957, Coleman returned to Salt Lake City to serve as Chief of Staff at Shriners Hospital for Children, a role he held until 1990. He took on Chair of the Division of Orthopedics for the UU later that year and served until 1981. He created the UU residency program in orthopedics, and later the UU fellowship in pediatric orthopedics. During his time on the board of the American Orthopedic Association (AOA), he gave a groundbreaking address about the need for sub-specialization. This address in 1978, many believe, was the impetus for the creation of fellowship training across the United States and internationally [3].

Educational impact

In addition to being a master surgeon, clinician, and leader in orthopedics, Coleman was internationally famous for his tremendous education acumen and Socratic teaching style.

Although there are endless examples of this style, one of the best was the beloved (and much feared) 0600 Friday morning “quiz” in pathology. From 1960 until just months before his passing in 2004, Coleman led this amazing conference. He procured radiographs from his years of work and selected four radiographic cases of orthopedic tumor pathology, along with 10 histologic slides, four of which matched the radiographic cases. Post Graduate Year (PGY) Four residents, until they became “higher council” in the spring to the incoming PGY 4s, were asked to describe the details of the radiographs in Coleman-approved language (e.g., *radiodense* or *radiolucent* never *osteodense* or *osteopenic*: “How can you tell the density of bone from shadows?”) and present a logical differential diagnosis. The goal was to match the correct diagnosis to the appropriate histological slide. All slides were described at the end and everyone hoped there would be only six left!

If the answer to a Coleman-generated question was unknown, he would often generously reply, “Well if you knew everything, you wouldn’t have to be here.” He would often assign a “two-and-a-half-minute talk” to the person unable to answer. These would be due the next Friday, often with citations. These “talks” harkened back to Coleman’s Mormon upbringing; Sunday school children or “Primary” as it was called in the church, were assigned a 2.5-minute talk on the scripture. When Coleman passed, I [KLC] was honored to give a 2.5-minute talk, at Coleman’s request, at his funeral, on behalf of all of us who learned so much from him. This was an incredible privilege.

There are many “Colemanisms.” He was brilliant, funny, and disarming, yet dogmatic, which was truly his gift. He was fanatical in his belief in the precision of language. No one would utter the word “subluxed.” If we did, Coleman would joke, “Well would you say *disloced*? Remember a *lux* is a unit of light, so if someone is subluxed, they’re dim!” *Internal* and *external *rotation were banned, as those terms imply* inside* or* outside *the extremity; only* inward* and* outward* rotation was allowed. A *short leg cast*? Never! It’s *below the knee*. All of us who learned from him still follow these rules. If you were doing well in surgery, “Good doctor, good doctor” was the highest of praise. Conversely, “Why don’t you just let me demonstrate this…” was a clear sign that you needed to improve your surgical skills.

Coleman’s scientific contributions

Coleman explored a plethora of topics and, in turn, produced numerous works including 88 articles, 16 book chapters, and two single-authored textbooks. These texts, titled *Congenital Dysplasia and Dislocation of the Hip* in 1978 and *Foot Problems* in 1983 are priceless. He developed lasting principles taught to aspiring orthopedic surgeons around the world and built upon those of other great researchers such as WV Anderson and Heinz Wagner. Although some of the science and knowledge have changed over time, the principles he taught remain steadfast: flexibility leads to soft tissue surgery, rigidity to bone correction; taking an entire transferred tendon across the midline is discouraged; true respect for your patients, students, and colleagues is the only way to practice.

Development of Novel Diagnostic Criteria and Management Standards

By personally examining over 1,000 Navajo infants for congenital hip dysplasia, Coleman unearthed multiple findings that have become well-established principles today. In his 1968 paper written in *Clinical Orthopaedics and Related Research*, Coleman found that while some cases of congenital hip dysplasia may spontaneously correct, others may persist despite negative physical examination findings. Moreover, he showed that dysplasia may progress to subluxation or complete dislocation of the hip, with or without precipitating physical findings [5]. Many of these findings led to the nomenclature of congenital hip dysplasia being changed to the modern *developmental dysplasia of the hip* that we use today.

**Figure 2:**
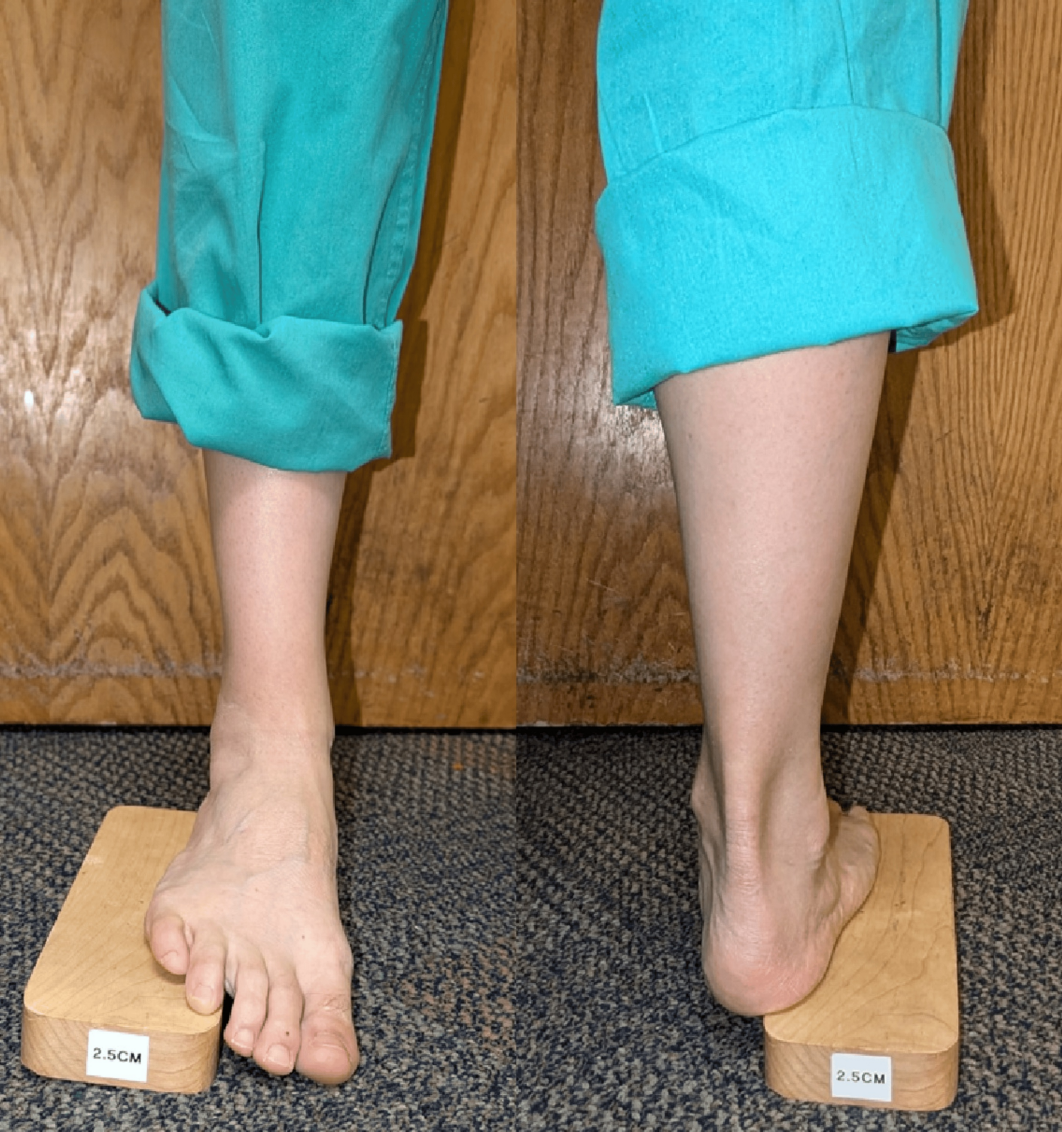
Anterior and posterior view of the Coleman block test. Original photo.

To localize the primary deformity in adult cavovarus feet, Coleman devised a test, now coined as the Coleman block test, that continues to inform the management of the condition. By placing the lateral portion of a patient’s foot on a 1-inch block while weight-bearing, the tester can discern the location and flexibility of the deformity (Figure 2). As explained in his 1977 paper in *Clinical Orthopedics and Related Research*, if the varus of the hindfoot corrects while weight-bearing on the block, the primary deformity is in the forefoot as the hindfoot is flexible. If flexibility is still present, then soft tissue surgery alone is indicated. Conversely, if the hindfoot varus does not correct, osteotomies or fusion may be necessary for effective treatment [6]. The Coleman block test was later evaluated by Foran et al who confirmed the test to be a reliable and inexpensive means of estimating the hindfoot alignment and flexibility in adult cavovarus feet [7].

Coleman also contributed to the widespread recognition of solitary lesser trochanter fractures being indicative of metastatic disease. He was the first to suggest the evaluation of metastatic disease in adults with this finding in his case series on the topic in 1984 [8].

Coleman’s academic achievements

Throughout his career, Dr. Coleman received many honors, including the Utah Governors’ Medal for Science and Technology, the Lifetime Achievement Award from the Utah Arthritis Foundation, and the posthumous Presidential Endowed Chair in Orthopedics at the University of Utah. He also was the first recipient of the Sherman S. Coleman humanitarian award of the Utah State Orthopedic Society and, finally, the Lifetime Achievement Award for the Pediatric Orthopedic Society of North America (POSNA) [1].

In addition to helping raise the UU orthopedic group from a division to a department (under his successor’s term), Dr. Coleman was president of every major orthopedic society in the United States: POSNA (which he helped create), the American Board of Orthopedics, the AOA, and the American Academy of Orthopedic Surgery.

## Conclusions

Dr. Coleman’s dedication to orthopedics, subspecialty training, orthopedic research, and education is unparalleled. He shaped the lives of decades of orthopedic residents and fellows who have continued to implement his special brand of teaching and orthopedic principles to others. His deep commitment to excellence and compassion in the care of his patients continues to inspire. His love of Utah, his family, and his orthopedic colleagues worldwide make us all better people.
